# Lower-limb internal loading and potential consequences for fracture healing

**DOI:** 10.3389/fbioe.2023.1284091

**Published:** 2023-10-12

**Authors:** Mark Heyland, Dominik Deppe, Marie Jacqueline Reisener, Philipp Damm, William R. Taylor, Simon Reinke, Georg N. Duda, Adam Trepczynski

**Affiliations:** ^1^ Julius Wolff Institute, Berlin Institute of Health at Charité—Universitätsmedizin Berlin, Berlin, Germany; ^2^ Department of Radiology, Charité—Universitätsmedizin Berlin, Berlin, Germany; ^3^ Centre for Muskuloskeletal Surgery (CMSC), Charité—Universitätsmedizin Berlin, Berlin, Germany; ^4^ Laboratory for Movement Biomechanics, ETH Zürich, Zürich, Switzerland

**Keywords:** internal bone loading, fracture fixation, femur, tibia, intramedullary nail, locking plate, *in vivo* loading, musculoskeletal modelling

## Abstract

**Introduction:** Mechanical loading is known to determine the course of bone fracture healing. We hypothesise that lower limb long bone loading differs with knee flexion angle during walking and frontal knee alignment, which affects fracture healing success.

**Materials and methods:** Using our musculoskeletal *in silico* modelling constrained against *in vivo* data from patients with instrumented knee implants allowed us to assess internal loads in femur and tibia. These internal forces were associated with the clinical outcome of fracture healing in a relevant cohort of 178 extra-articular femur and tibia fractures in patients using a retrospective approach.

**Results:** Mean peak forces differed with femoral compression (1,330–1,936 N at mid-shaft) amounting to about half of tibial compression (2,299–5,224 N). Mean peak bending moments in the frontal plane were greater in the femur (71–130 Nm) than in the tibia (from 26 to 43 Nm), each increasing proximally. Bending in the sagittal plane showed smaller mean peak bending moments in the femur (−38 to 43 Nm) reaching substantially higher values in the tibia (−63 to −175 Nm) with a peak proximally. Peak torsional moments had opposite directions for the femur (−13 to −40 Nm) versus tibia (15–48 Nm) with an increase towards the proximal end in both. Femoral fractures showed significantly lower scores in the modified Radiological Union Scale for Tibia (mRUST) at last follow-up (*p* < 0.001) compared to tibial fractures. Specifically, compression (*r* = 0.304), sagittal bending (*r* = 0.259), and frontal bending (*r* = −0.318) showed strong associations (*p* < 0.001) to mRUST at last follow-up. This was not the case for age, body weight, or localisation alone.

**Discussion:** This study showed that moments in femur and tibia tend to decrease towards their distal ends. Tibial load components were influenced by knee flexion angle, especially at push-off, while static frontal alignment played a smaller role. Our results indicate that femur and tibia are loaded differently and thus require adapted fracture fixation considering load components rather than just overall load level.

## 1 Introduction

Beside biological challenges, such as immune competence of the patient (Kolar et al., 2010; Reinke et al., 2013; Borgiani et al., 2019; Schlundt et al., 2019; Bucher et al., 2022; Duda et al., 2023; Jahn et al., 2023; Malhan et al., 2023), the mechanical boundary conditions can also impact fracture healing (Kassi et al., 2001; Epari et al., 2007; Ode et al., 2014; Santolini et al., 2015). Mechanical cues are known to control tissue regeneration and especially bone tissue regeneration as a model system of regeneration (Drzeniek et al., 2021; Knecht et al., 2021), and mechanical overloading may disrupt early callus healing. In addition to the direct mechanical environment, patient anatomy, activity, fracture geometry, and fixation (applied osteosynthesis) are factors known to impact fracture healing outcome.

Loading strongly determines the course and outcome of fracture healing (Seebeck et al., 2005), which has been acknowledged as one out of four key factors influencing healing outcome: osteogenic cells, osteoinductive mediators, osteoconductive matrix, and mechanical loading stability—“the diamond concept” (Giannoudis et al., 2007; Giannoudis et al., 2008; Willie et al., 2010; Giannoudis et al., 2014; Giannoudis et al., 2015).

Mechanical loads between bones, as well as across localisations within a single bone, are expected to differ, for instance due to attachment sites and activation patterns of muscles, or patient anatomy. The variation of these forces and moments between patients and localisations and how the specific loading may be associated with alterations in healing success is still speculative. Delayed healing, i.e., no or only little signs of healing for 3–6 months, or non-union fractures, i.e., absent healing for more than 6–9 months, not only affect patients, but also results in a high economic burden (Kanakaris and Giannoudis, 2007). Therefore, greater knowledge of fracture healing and further improvement in clinical settings is essential.

Only few studies have tried to quantify the mechanical boundary conditions acting at a fracture location (Schwarzenberg et al., 2020; Augat et al., 2021). To define the local mechanical boundary conditions for successful fracture healing, it is necessary to extend the knowledge about local forces and moments acting *in vivo* in long bones (Duda et al., 1997a; Duda et al., 1997b; Taylor et al., 2004; Bergmann et al., 2016; Dreyer et al., 2022) and how they interact with the selected fixation of fragments (Märdian et al., 2021).

With the present work, we aimed to determine the internal loads acting within the lower limb long bones (femur and tibia) during walking using a validated musculoskeletal modelling approach (Trepczynski et al., 2012; Trepczynski et al., 2018), and to associate them with fracture healing outcome. We hypothesise that changes in patient kinematics, represented by knee flexion angle, and anatomical alignment, represented by frontal plane knee alignment, would allow internal femoral and tibial loading to be assessed, and thus make it possible to determine the mechanical boundary conditions for successful fracture healing. Such understanding of the mechanical boundary conditions and the parameters influencing them appears especially important for guiding the selection of a fracture fixation that is capable of appropriately counteracting and stabilising the specific load components (forces and moments) and thereby to ensuring beneficial mechanical boundary conditions for successful healing, independent from the long bone affected or the specific location of a given fracture.

## 2 Materials and methods

**FIGURE 1:**
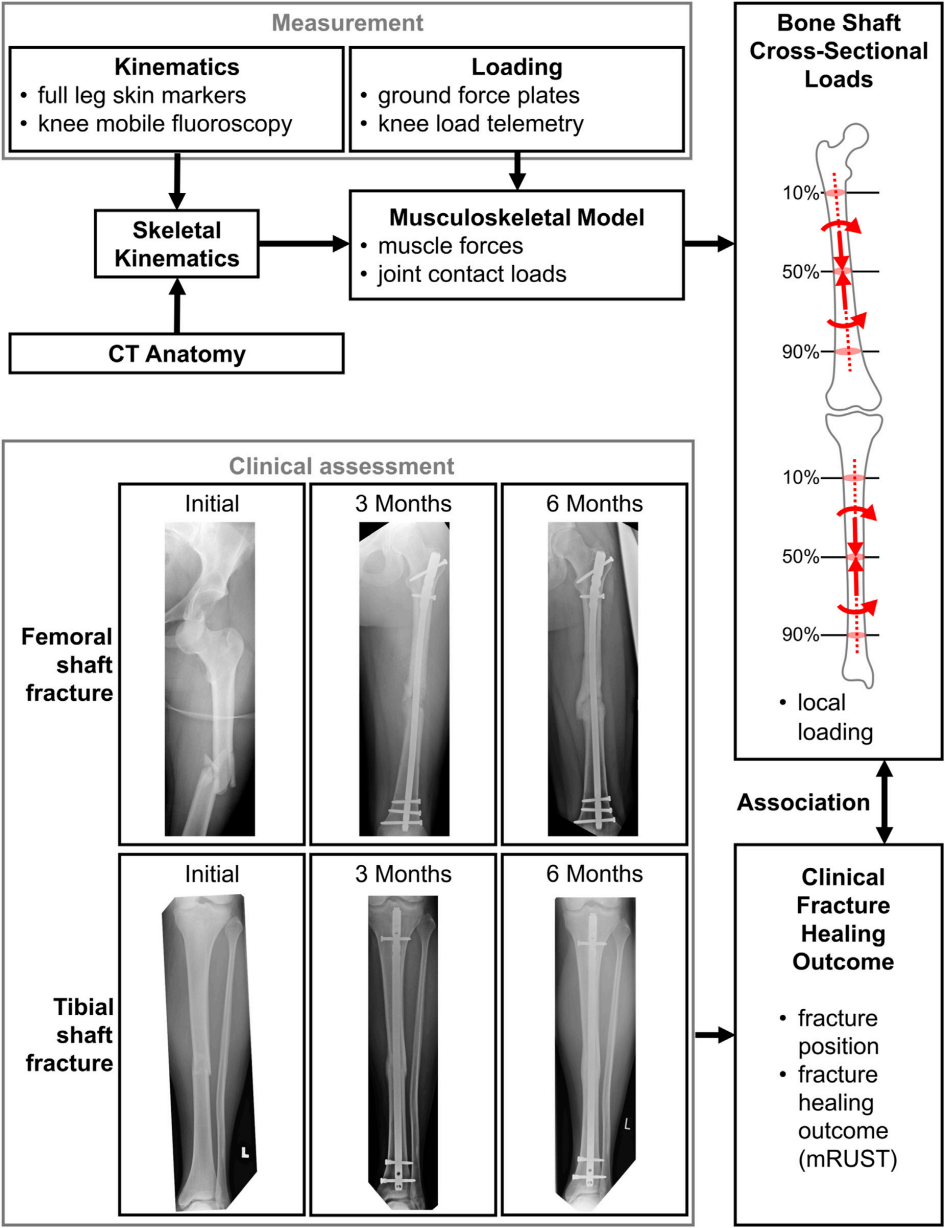
The measurement and modelling pipeline to assess internal bone loads, which are thought to be associated with the clinical outcome.

In this study, we utilised our validated musculoskeletal *in silico* models, which are based on *in vivo* measured knee loads from patients equipped with instrumented knee implants. This allowed us to constrain the model solutions to realistic *in vivo* loading conditions. An *in vivo* fluoroscopic assessment allowed us to determine patient-specific kinematics. These internal forces are considered to represent the mechanical boundary conditions under which a fracture fixation would affect bone healing in extraarticular fractures of the lower limb (Figure 1).

### 2.1 Musculoskeletal modelling

Our musculoskeletal modelling was based on the 3D patient-specific anatomy reconstructed from CT imaging data, ground reaction forces, and kinematic input from gait analysis (Trepczynski et al., 2012; Trepczynski et al., 2018), and allows muscle forces and resulting joint contact loads to be estimated for the whole leg. The musculoskeletal analysis used in this investigation was based on the CAMS-Knee Dataset (https://cams-knee.orthoload.com/), which provides a comprehensive combination of *in vivo* measured inputs for this type of model (Taylor et al., 2017). Six patients (5 male, 1 female), aged 74 (65–80) [mean (range)] years, with body-mass of 89 (67–101) kg, and body-height of 172 (165–175) cm (Supplementary Table S1 or https://cams-knee.orthoload.com/subjects/) were previously implanted with instrumented knee implants that allowed the *in vivo* tibio-femoral (TF) contact forces and moments (Heinlein et al., 2007) to be telemetrically measured. The six patients (with the codes K1L, K2L, K3R, K5R, K7L, and K8L) were asked to perform several repetitions of walking at self-selected speeds in a laboratory setting, yielding a minimum of 5 gait phases of stance for each patient that were suitable for further analysis. The internal forces were recorded synchronously with the internal knee kinematics, which were captured using a mobile video-fluoroscope (List et al., 2017).

Subsequently, musculoskeletal modelling was performed for each patient to estimate the muscle and joint contact forces in the lower limb as described previously (Trepczynski et al., 2018; Kneifel et al., 2023), briefly outlined here: Lower limb kinematics were derived from skin marker motion (Trepczynski et al., 2012), and combined with the functional flexion knee axis derived from the fluoroscopic TF kinematics (Taylor et al., 2010; Ehrig et al., 2011; Heller et al., 2011), serving as input to an inverse dynamics approach. The subsequent muscle optimisation was based on minimising the sum of muscle stresses squared, but was also constrained to match the magnitude of the TF contact force measured *in vivo* to within 5%.

### 2.2 Cross-sectional loads acting within the lower limb long bones

**FIGURE 2:**
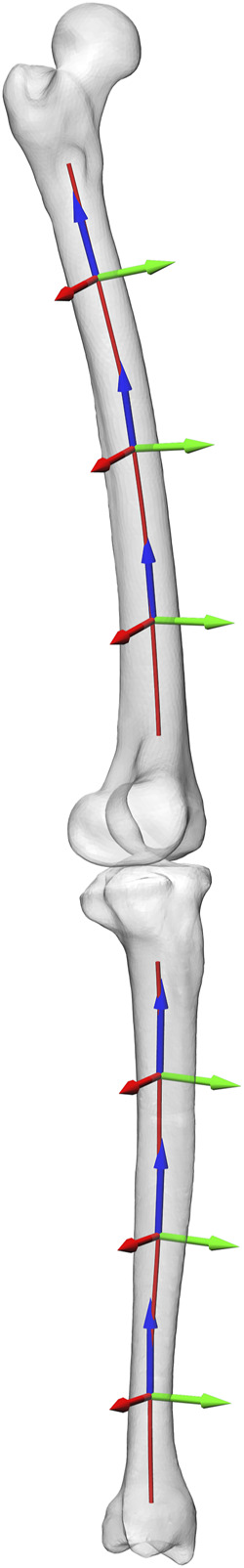
The shaft centroid lines of femur and tibia along which the bone cross sectional loads were evaluated, with local coordinate systems shown at 20%, 50%, an 80% shaft length.

The muscle and joint contact forces yielded by the musculoskeletal model of the patients from the CAMS-Knee Dataset were then used to compute the local bone loading along the centroids of the lower long bone shafts. The curved shaft centroids were generated from the patient-specific bone geometry taken from CT data using the auto skeleton module of Amira (Thermo Fisher Scientific Inc.) (Figure 2). The proximal end of the femoral shaft was defined at the height of the trochanter minor [corresponding to 16% ± 1% (mean ± SD) of the total bone length from proximal], while the distal end was placed half of the inter-epicondylar distance above the inter-epicondylar midpoint (85% ± 1% bone length). For the tibia, the proximal shaft end was defined at the height of the tibial tuberosity (13% ± 1% bone length), while the distal end was defined half of the inter-malleoli distance above the inter-malleoli midpoint (93% ± 0% bone length). The local bone loads, resulting from the joint contact forces and the spanning musculature, were determined along the centroid curve in each lower limb bone in 5% steps of its total length. At each evaluation point (EP) a local coordinate system was defined (Figure 2), with the axial base vector along the local centroid tangent defining the local transverse plane of the shaft. The base vectors in the local transverse plane were based on the medio-lateral direction defined from the epicondyles for the femur and tibial plateau compartment centres for the tibia. The components of the cross-sectional loading in each bone were based on this local coordinate system, with the moments acting around the EP at the centroid locations. Within each bone and for each shaft location, load components were determined as loading range during the stance phase in each patient and repetition. To allow for comparison across patients, all forces from the analysis were normalised to multiples of bodyweight (BW), while all moments were normalised to multiples of bodyweight times metre (BWm). The adapted sign convention ensured that load values for left and right legs were consistent.

Linear regression was applied to investigate the relationships between the extremal cross-sectional loads within both bones and the hip-knee-ankle (HKA) angle from static frontal plane radiographs, as well as the knee flexion at 20%, 50%, and 80% of the stance phase, which approximately corresponds to the time points of the early and late joint loading peaks (if present), and their intermediate time point.

### 2.3 Clinical assessment of extra-articular healing in the femur and tibia using a retrospective analyses of fracture healing outcomes

The inclusion criteria for the retrospective analyses of fracture healing was a patient age of minimal 18 years, and patients undergoing surgery for an extra-articular fracture of a long bone of the lower extremity (femur or tibia) performed between January 2005 and April 2022. For inclusion, patient fracture X-rays had to be taken in two perpendicular planes, one taken pre- and one post-surgery plus at least one follow-up time point.

The exclusion criteria ruled out patients with critical clinical conditions at the time of the operation (e.g., unstable circulatory conditions, not fit for surgery and/or lack of consent of treatment), pregnant and lactating patients, persons who were not legally competent, and fractures involving articulation, including proximal femur and femoral neck fractures. We excluded cases with insufficient imaging data quality, lacking or inadequate documentation, or lack of follow-up imaging.

Overall, a total of 4,841 cases were screened with treatment performed at our local trauma centre, resulting in 190 fractures from 171 patients, of which 154 patients had only a single fracture, 15 had 2 fractures, and 2 had 3 fractures. Finally, closer inspection revealed that some of those cases still contained fractures reaching into the joint articulations and had to be excluded, resulting in a total of *N* = 178 fractures used for analysis (Figure 1; Table 1).

**TABLE 1:** Fracture patient demographics [mean (SD: standard deviation); range or absolute numbers], and treatments.

Fracture location	Age	Sex female: male	Weight	Fixation Nail: Plate
All (*N* = 178)	43.0 (SD 15.4) years; 18–84 years	55: 122 (1 undetermined)	*N* = 92 81.7 (SD 15.5) kg; 52–118 kg	135: 43
Femur (*N* = 57)	40.4 (SD 16.4) years; 18–84 years	17: 40	*N* = 27 81.4 (SD 15.0) kg; 60–110 kg	41: 16
Tibia (*N* = 121)	44.3 (SD 14.8) years; 18–78 years	38: 82 (1 undetermined)	*N* = 65 81.9 (SD 15.8) kg; 52–118 kg	94: 27

Localisation of fractures was recorded as a relative position along the long bone axes between a proximal to a distal location and cases were grouped in steps of a fifth of the total long bone lengths (i.e., 10%/30%/50%/70%/90% of bone length). Further, fracture cases were separated according to the treatment method used with either intramedullary nailing or plate fixation.

Healing success was assessed using the modified Radiographic Union Score for Tibia, or modified Radiological Union Scale for Tibia (mRUST) fractures, an established and validated score that judges the bridging and fracture line (from 4—no bridging to 16—complete consolidation at each cortical and two planes) of the fracture of tibia or femur by a radiologist blinded for time points and clinical information (Fiset et al., 2018; Mitchell et al., 2019; Plumarom et al., 2021; Alentado et al., 2022; Schmidt et al., 2022).

### 2.4 Statistical evaluation

The mRUST at the last follow-up time point was compared statistically in femur versus tibia fractures, for nail versus plate fixation as well as along the fracture localisations within each long bone (Mann-Whitney-U-Test). Finally, we combined the model-based mean of local peak loading over all modelled subjects with the localisation of the fracture (inter- and intraosseous: comparison of healing outcome between femur and tibia, but also localisations within the same bone of the retrospective clinical fracture patients), and tested for associations (Pearson correlation) of the expected local load, localisation of fracture within the bone, age, and body weight, with the healing outcome (mRUST at last follow-up).

## 3 Results

### 3.1 Joint contact force

**FIGURE 3:**
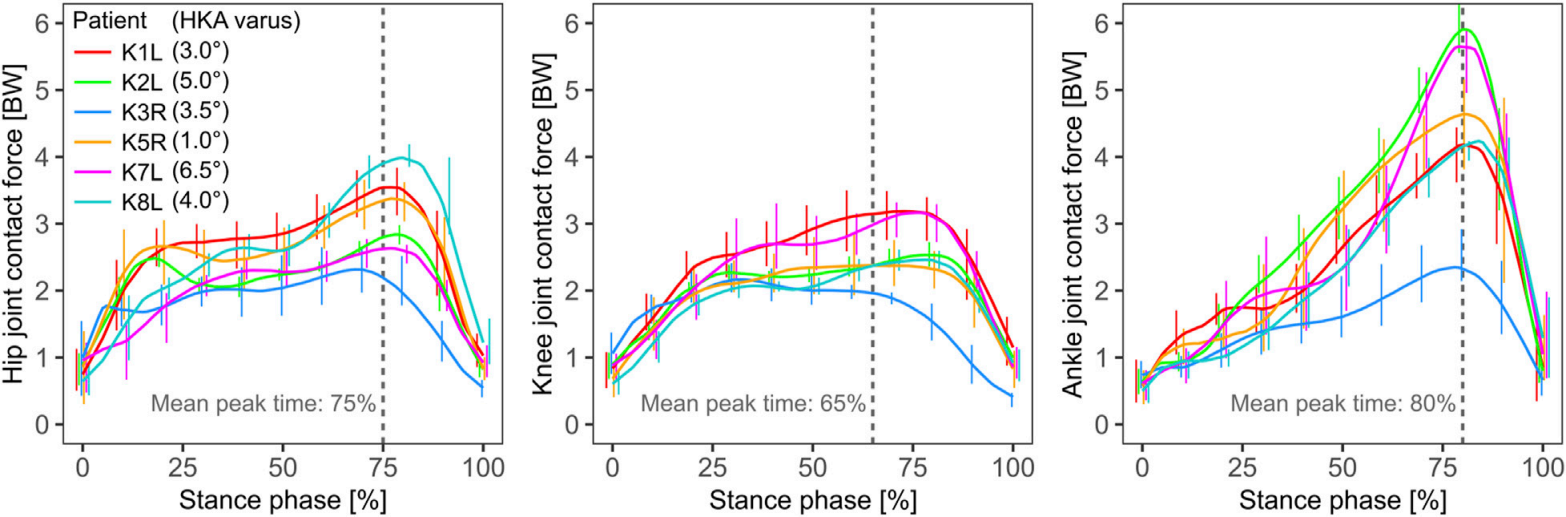
The joint contact force at the hip, knee and ankle from left to right respectively, predicted by the model, with the knee contact force magnitude constrained to match the *in vivo* measured force. The mean time point of the peak force is marked with a dashed grey line.

The joint contact force (JCF) for hip and knee followed similar patterns and reached similar peak levels of ∼2–4 BW, usually occurring in the late stance phase (Figure 3). The mean peak hip JCF ranged from 1,751 (patient K7L) to 3,672 N (K1L), while the mean peak knee JCF ranged from 1,946 (K8L) to 3,305 N (K1L). For the ankle, the mean peak JCF was always in the late stance phase, where it showed a larger variation across patients than those at the other two joints, ranging from 2,403 (K3R) to 5,343 N (K2L). In particular the substantially lower ankle JCFs in K3R were observed to be associated with a different push-off pattern in this patient with engagement of the entire foot instead of just the forefoot as in the other five patients.

### 3.2 Cross-sectional loads within femur and tibia

**FIGURE 4:**
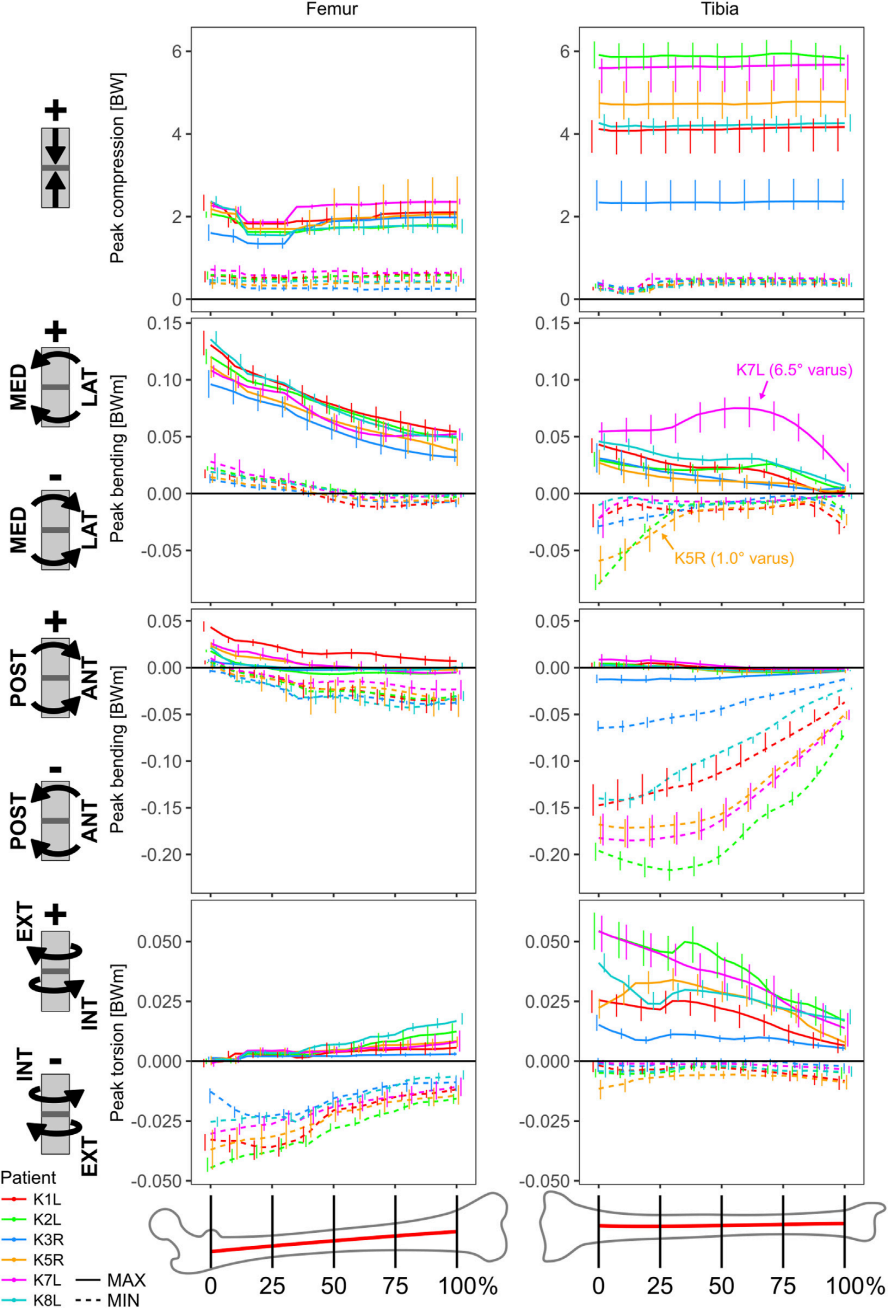
Cross sectional loading range during the stance phase of walking as a function of the relative location along the extra-articular section of bone shaft.

The peak compressive loads in the tibia were more than two times greater than in the femur, but roughly constant along the shaft for both bones (Figure 4). While the mean peak compression at 50% shaft length for the femur ranged from 1,330 (K8L) to 1,936 N (K1L), compression in the tibia ranged from 2,299 (K3R) to 5,224 N (K2L). Here again, the distal tibial shaft loading in patient K3R appeared related to the lower ankle JCFs, possibly related to their distinct push-off pattern engaging the whole foot in a flat contact while all others exhibited a forefoot contact pattern at push-off.

In the frontal plane, the mean peak bending moments were greater for the femur than for the tibia, and increased towards the proximal ends, where for the femur they ranged from 71 (K7L) to 130 Nm (K1L), while for the tibia they ranged from 26 (K5R) to 43 Nm (K1L). In the sagittal plane, the mean peak bending moments for the femur overall remained between −38 (K3R) and 43 Nm (K1L), while at the proximal end of the tibial shaft values reached −63 (K3R) to −175 Nm (K2L).

The peak torsional moments had opposite directions for femur versus tibia and increased in magnitude towards the proximal ends in both bones. Torsional moments for the femur ranged from −13 (K3R) to −40 Nm (K2L), while they ranged from 15 (K3R) to 48 Nm (K2L) for the tibia.

### 3.3 Different impact of static frontal knee alignment versus dynamic knee flexion on bone loading

**FIGURE 5:**
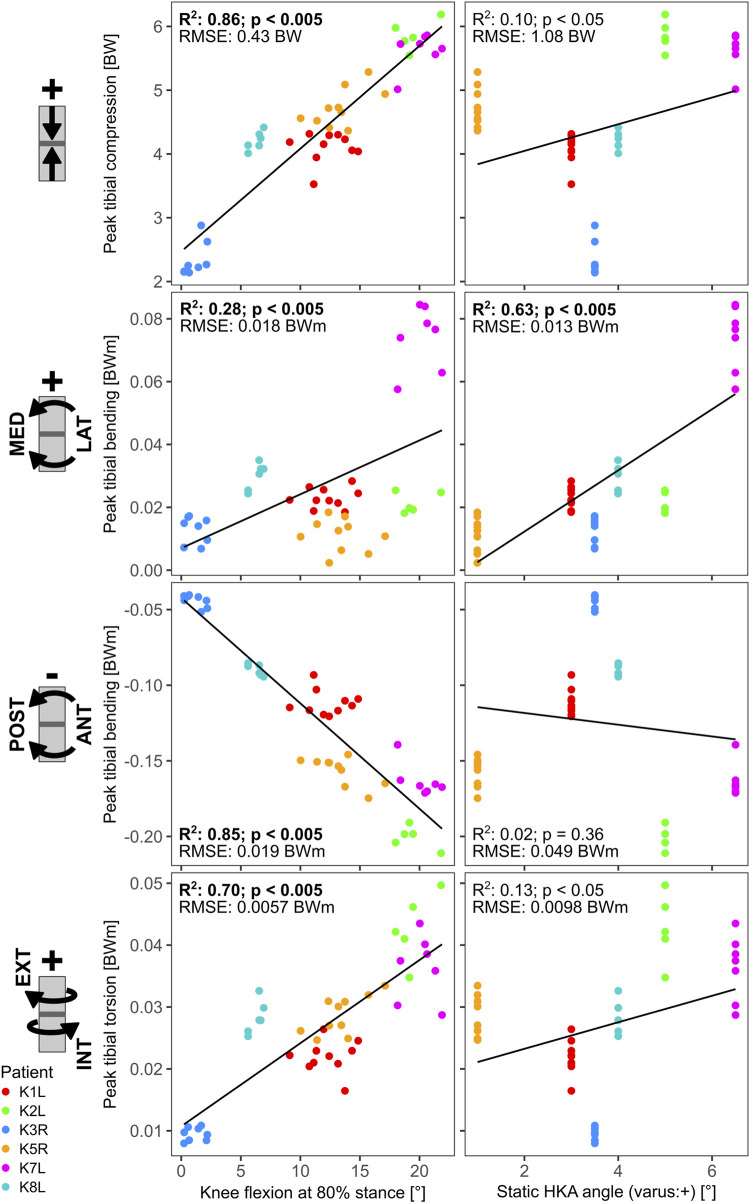
Linear regressions between late stance knee flexion (at 80% stance), static HKA angle and the tibia loading at 50% shaft length.

The linear regressions between static frontal plane alignment, knee flexion during stance, and the dominant cross-sectional loading along the long bones showed only weak relationships for the femur, with *R* ^2^ values below 0.5. For the tibia however, several strong relationships were found (Figure 5). For the mid-shaft of the tibia, the knee flexion at 80% of the stance phase had strong relationships with the peak compression (*R* ^2^ = 0.86), sagittal plane bending (*R* ^2^ = 0.85), and torsion (*R* ^2^ = 0.70). The static HKA angle on the other hand, showed only relevant correlation with the frontal plane bending (*R* ^2^ = 0.63).

### 3.4 Comparing long bone shaft loading with fracture healing outcome

**FIGURE 6:**
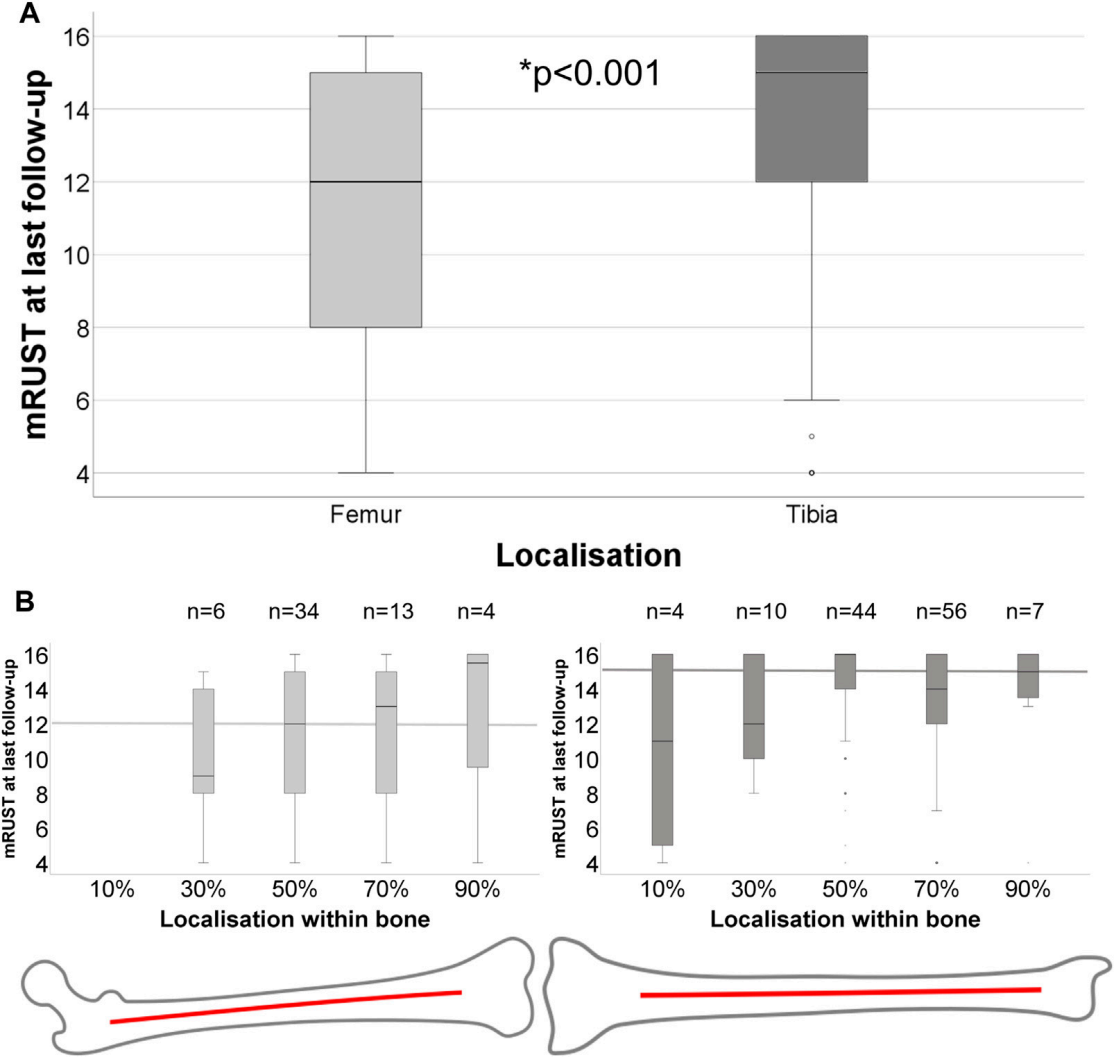
**(A)** Healing outcome at last follow-up for different bones, femur (*n* = 57) and tibia (*n* = 121), differs significantly (Mann-Whitney-U: *p* < 0.001). **(B)** Healing outcome according to detailed localisation within the bone from proximally to distally for femur and tibia. Median mRUST at last follow-up of each bone is marked with thick lines.

In our retrospective, clinical analyses, femoral fractures showed significantly decreased mRUST at last follow-up (*p* < 0.001, Mann-Whitney-U) compared to tibial fractures (Figure 6A). Independent nail fixation, which was much more prominent in this sample (Table 1), showed more consistent healing with less variability compared to plate fixations in the distal part of the bones for femur fixation (Supplementary Figure S1), but with no detectable differences in mRUST between nail and plate fixation. Despite the large variability of healing outcomes, more distal fractures within the bones appeared to show higher mRUST values at last follow-up (Figure 6, not significant). Different local loading (as assessed by the modelling) showed significant (*p* < 0.001) associations with mRUST at last follow-up, in detail: compression (*r* = 0.304), sagittal plane bending (*r* = 0.259), and frontal plane bending (*r* = −0.318, negative correlation). However, mRUST was not associated to age, body weight, or facture localisation along the long bone axis alone (Table 2).

**TABLE 2:** Pearson correlation of model-based mean loading at the respective fracture location (according to bone type and intra-osseous fracture location), and Pearson correlation of localisation within bone, age, weight with mRUST at last follow-up for femur and tibia (pooled and separately).

	Compression	Sagittal bending	Frontal bending	Torsion	Localisation within bone	Age	Weight (*n* = 92)
Pearson correlation coefficient r with mRUST (tibia and femur pooled together, *N* = 178)	**0.304**	**0.259**	**−0.318**	0.090	0.141	−0.108	−0.167
*p*-value	**<0.001**	**<0.001**	**<0.001**	0.233	0.061	0.149	0.111
Pearson correlation coefficient r with mRUST Femur (*N* = 57)	0.146	0.159	−0.170	−0.167	0.170	−0.048	−0.199
*p*-value	0.279	0.238	0.207	0.214	0.206	0.725	0.321
Pearson correlation coefficient r with mRUST Tibia (*N* = 121)	0.000	−0.037	−0.055	−0.034	0.095	**−0.220**	−0.185
*p*-value	0.998	0.690	0.549	0.715	0.300	**0.015**	0.140

Statistically significant Pearson correlations (*p* < 0.05) are marked in bold.

## 4 Discussion

We aimed to reliably determine the internal loading conditions acting within the tibia and femur by employing *in vivo* measurements and musculoskeletal modelling to provide a basis for optimised treatments. Further, we aimed at associating these loads to a retrospective analysis of fracture healing outcome in extra-articular fractures of these bones. Apparently, fracture healing outcome—as reflected by mRUST—appears to be associated more strongly with the specific load components than with age, sex, or bodyweight of the patients.

Local load components that act during gait showed a positive association with healing as seen for compression or sagittal plane bending, but also a negative association with frontal plane bending (Table 2). This has already been indicated in a previous animal study, where just different placement of fixators could already control for the effect of frontal plane bending, by changing mediolateral bending stiffness, resulting in much better healing with a stiffer construct in the frontal plane (Epari et al., 2007). At the same time, moderate stiffness in compression and sagittal bending yielded the best healing results (Epari et al., 2007). Thus, high fixation stiffness is only reasonable against shear, torsion and frontal plane bending, while the other fixation stiffness components should be moderately rigid and adapted to the requirements of patient weight and activity, as well as fracture location.

During gait, the tibia appeared more compressed than the femur, mainly associated with plantar-flexor muscle activation during late stance phase particularly at push-off. As expected, the mean bending moments in the frontal plane were greater for the femur than for the tibia, and decreased towards each long bone’s distal end (Duda et al., 1997b; Duda et al., 2002; Kutzner et al., 2010). In the sagittal plane, mean bending moments for the femur overall remained moderate, while for the proximal end of the tibial shaft they reached high values, decreasing towards its distal end.

Torsional moments had opposite directions for the femur and tibia, again with decreases in magnitude towards the distal ends. The hip joint loads were consistent with direct measurements (Bergmann et al., 2016; Damm et al., 2017; Palmowski et al., 2021). The femoral loads were consistent with previous assessments of internal loads (Duda et al., 1997b). Overall, we saw higher loads in the proximal parts of the bones, except for the load component of compression, which was similar all over the tibia and femur, but at a higher level in the tibia.

When the mean peak loads derived from modelling were compared to healing outcomes for the different fracture locations along the long bone axis of the tibia or femur, load components showed stronger association to fracture healing outcomes than age, body weight, or sex. Also, the fracture localisation within a long bone did not show a strong correlation to bone healing outcome (Table 2). Despite the higher load magnitudes in the tibia during normal walking as derived from the modelling, we see higher mRUST at last follow-up in the tibia compared to the femur, which indicates that compressive loading is not necessarily detrimental to healing. Interestingly, another study also found a higher prevalence of non-union cases in femoral (54%) versus tibial (34%), or other fractures (11%) that were treated in a revision for bone repair (Giannoudis et al., 2015). Importantly, we did not find significant differences in healing outcomes with different types of nailing versus plating in either the tibia or femur (Supplementary Figure S2).

We hypothesised that changes in knee flexion angle and frontal knee alignment lead to altered internal muscle and joint loads, which affect fracture healing. Local bone loading can be influenced not only by different activities (Kutzner et al., 2010; Schwachmeyer et al., 2013; Bergmann et al., 2016; Haffer et al., 2021), but also by their execution (e.g., gait pattern, speed), (Trepczynski et al., 2014; Trepczynski et al., 2018). Confirming our hypothesis, we could show that late stance knee flexion had strong relationships with the peak compression, sagittal plane bending, and torsion in the tibia, which implies that only certain loading components can be influenced by the patients. The static frontal plane alignment correlated with peak frontal plane bending in the tibia. Effects of changes in knee flexion angle and frontal knee alignment on the internal loading conditions were much weaker within the femur. This means loading could potentially be influenced to a much greater degree and with multiple options at the tibia compared to the femur.

In the past, worse healing at certain locations was often attributed to worse perfusion or fewer available osteogenic cells at locations such as the more distal parts of the femur and tibia (Santolini et al., 2014; Santolini et al., 2015; Wildemann et al., 2021). We did not assess differences in perfusion or other biological differences of the patients in the retrospective study. However, in our assessment, the more distal fractures appear to have a more consistent healing (Figure 6B), but this was not a significant improvement due to the low number of cases and high variability, which might be associated with the fixation type and bone (Supplementary Figures S1, S2). A limitation of the present study is that we employed loads in otherwise healthy “normal gait” settings of patients after TKA as a reference for the forces and moments acting in long bones after fracture. These loads during early rehabilitation might well differ (Seebeck et al., 2005), however, or other loading conditions such as chair rise might be more relevant for success or failure of long bone healing. A limitation of our retrospective analysis of 4,841 fracture healing cases was that only 178 fracture cases qualified for being included and also follow-up periods were different between patients, as well as the last time point of follow up was variable. In the future, we aim to examine prospective fracture healing studies with dedicated study documentation to ensure a more comprehensive data basis and allow causal relationships to be elucidated between mechanical limb loading and healing outcome. For the fracture patients the evaluations were performed on standard clinical radiographs, which did not always cover the entire bone, making the relative localisation an estimate. In the presented retrospective analysis of extra-articular fractures, we had mainly young (<40 years) or middle-aged (40–60 years) patients included with a higher incidence in young males with tibial fractures. We missed the otherwise frequently indicated group of elderly women above 60 years (Court-Brown and Caesar, 2006) in our analyses. Although we found a bimodal distribution in our data, the two modes exist rather between young and middle-aged patients with a similar distribution for men and women. We conclude that in our retrospective assessments, such selection effects clearly result in bias and we see the need for more prospective analyses, especially for geriatric, frailty fractures. We found a significant negative correlation between age and mRUST at last follow-up for the tibia fractures (Table 2), and suspect that this poorer healing outcome with age would also be present in a larger cohort of femoral fractures. However, long bone loading within a lower limb appears to be modulated by patient knee flexion during push-off phase in walking. In our assessment, healing of femur fractures did not worsen with age. Moreover, nail fixation was much more prevalent than plate fixation. We did not control for fixation choice, nor normalise the fracture patterns, such as fracture classifications, slope, orientation, or degree of comminution. Also, we did not control for individual muscle status (Damm et al., 2018; Damm et al., 2019; Winkler et al., 2023). Muscle activity assumes ideal muscle use to match the measured kinematics and knee joint loading. Geriatric or pain-induced muscle activation and gait patterns will be different, but it is not age that mostly determines different activation patterns, but rather extensive muscle damage (Damm et al., 2018; Damm et al., 2019; Winkler et al., 2023) or neurological co-morbidities which were not considered in this approach. We strive to investigate early rehabilitation gait patterns and detailed age- and muscle-status related changes in kinematics and loading in more detail in fracture patients in the future.

In summary, our results advocate the importance of specific loading components on fracture outcome rather than overall load magnitude alone. We conclude that local loading components are differently associated with healing outcome in this assessment for a specific patient cohort: While higher compressive loads and bending in the sagittal plane were correlated to higher fracture healing/bridging scores at last follow-up, higher moments in the frontal plane were negatively correlated with healing outcomes. More detailed assessments of individual fracture cases, considering the patient-specific kinematics (gait speed, flexion angles) and anatomy (alignment) to achieve even more accurate local loading assessments, but also considering the fracture pattern and local strain at the fracture zone, could further elucidate the context of loading and mechanical tissue stimulation for fracture healing. Well-controlled, adapted mechanical loading may represent one integral part to the solution for a coordinated fracture healing. The loading patterns during walking for lower limb long bones reported from our validated musculoskeletal model analyses could be used in further studies to identify “ideal fixation configurations” to optimize fracture treatment in the future.

## Data Availability

The datasets presented in this article are not readily available because restrictions apply to the availability of the implant geometry data, which were used under license for the current study, and so are not publicly available. Requests to access the datasets should be directed to https://cams-knee.orthoload.com/data/data-download.
