# Supplementary material for: Lower-limb internal loading and potential consequences for fracture healing
Source: Front Bioeng Biotechnol. 2023 Oct 12;11:1284091. doi: 10.3389/fbioe.2023.1284091 (PMC10602681; doi:10.3389/fbioe.2023.1284091)
Supplement: Supplementary file 1 [file DataSheet1.PDF]

Supplementary Material

1 Supplementary Tables

**Subject**	**K1L**	**K2L**	**K3R**	**K5R**	**K7L**	**K8L**
Gender	m	m	m	m	f	m
Age	69	78	77	66	79	70
Body mass [kg]	101	91	100	96	67	79
Height [cm]	175	169	173	174	165	175
Time post-op [months]	87	84	76	69	67	64
Tibio-femoral angle [degree]	3.0 varus	5.0 varus	3.5 varus	1.0 varus	6.5 varus	4.0 varus
Posterior slope [degree]	5	11	10	7	7	11

Supplementary Table 1. Overview of patients whose gait was measured for musculoskeletal modelling (Information concerning this data and documentation is available at https://cams-knee.orthoload.com/).

**Peak compression [N]**	**FEMUR max Fz**	**Change towards distally (%)**	**TIBIA max Fz**	**Change towards distally (%)**
10%	1787 (229)	REF	4126 (996)	REF
50%	1886 (424)	+5.5	4140 (991)	+0.3
90%	2001 (507)	+12.0	4179 (989)	+1.3
**Peak frontal bending [Nm]**	**FEMUR max My**		**TIBIA max My**	
10%	97.0 (17.3)	REF	36.2 (6.5)	REF
50%	65.9 (13.3)	-32	27.9 (14.2)	-23
90%	48.3 (9.7)	-50	12.1 (11.2)	-67
**Peak sagittal bending [Nm]**	**FEMUR min Mx**		**TIBIA min Mx**	
10%	9.2 (4.5)	REF	139.3 (46.0)	REF
50%	30.4 (10.7)	+230	117.9 (52.2)	-15
90%	38.1 (10.4)	+314	57.1 (30.9)	-59
**Peak torsion [Nm]**	**FEMUR min Mz**		**TIBIA max Mz**	
10%	29.7 (9.3)	REF	31.5 (12.6)	REF
50%	20.1 (4.8)	-32	27.7 (10.8)	-12
90%	13.2 (4.3)	-56	15.4 (5.6)	-51

Supplementary Table 2. Mean (standard variation) of extremal loading during the stance phase of walking as function of the relative location along the extra-articular part of bone shaft (proximal to distal).

Model	A	B	C	R^2	RSME
**TIBIA at 50% shaft**					
max Fz = A + B * F80	2.47	0.16	-	**0.86**	0.43
max My = A + B * HKA	-0.0072	0.010	-	**0.63**	0.013
min Mx = A + B * F80	-0.042	-0.007	-	**0.85**	0.019
max Mz = A + B * F80	0.011	0.0013	-	**0.70**	0.0057
**FEMUR at 10% shaft**					
min Mz = A + B * F80 + C * HKA	-0.026	-0.00086	0.0017	**0.67**	0.0039
min Mz = A + B * VEL + C * HKA	-0.0065	0.035	0.0013	**0.70**	0.0027

Supplementary Table 3. Results of linear regressions between anatomy, kinematics and peak bone loading. HKA [°]: hip-knee-angle]; F80 [°]: knee flexion at 80% stance phase; VEL [m/s]: mean forward speed during stance phase; Mx [BWm]: peak bending in the sagittal plane (negative: ends bent to posterior); My [BWm]: peak bending in the frontal plane (positive: ends bent to medial); Fz [BW]: peak compression.

2 Supplementary Figures

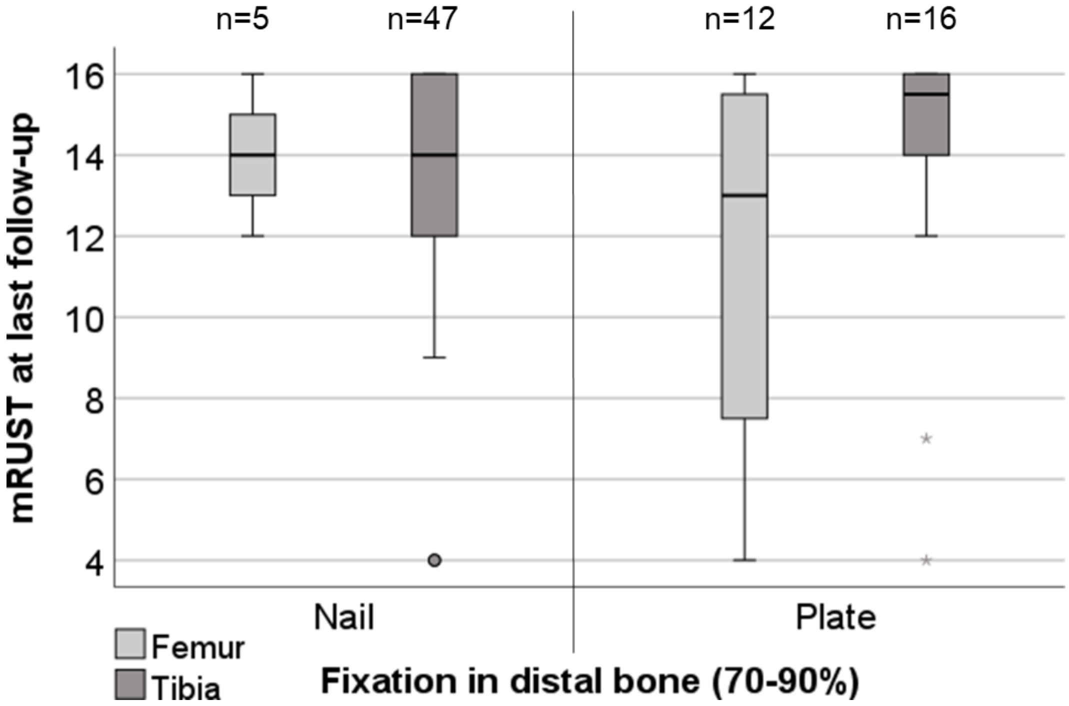

Supplementary Figure 1: Healing outcome for distal bone fractures according to treatment and bone.

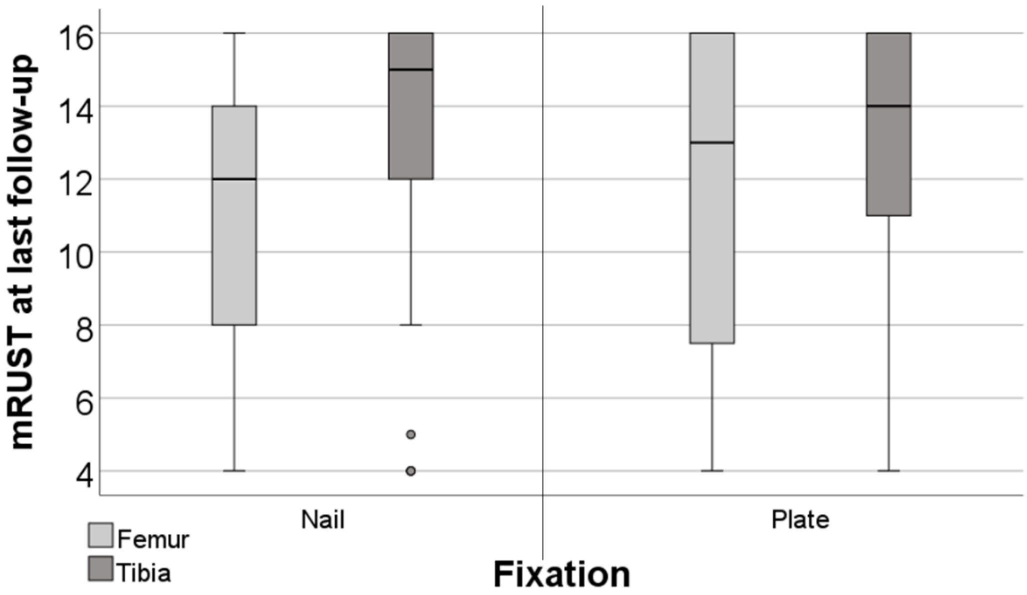

Supplementary Figure 2: Healing outcome for fractures according to treatment and bone.
